# Comprehensive Rehabilitation for Autosomal Dominant Retinitis Pigmentosa: A Case Report

**DOI:** 10.1155/crop/4856296

**Published:** 2025-09-01

**Authors:** Joshua L. Robinson

**Affiliations:** Department of Ophthalmology and Visual Sciences, Vanderbilt University Medical Center, Nashville, Tennessee, USA

## Abstract

A 62-year-old female with retinitis pigmentosa presented for a low vision rehabilitation evaluation. An updated spectacle prescription, filters, and task lighting were beneficial, but the patient was left with outstanding needs. She noted that she had lost her independence and felt trapped within her own home with nobody around who could fully understand her situation. Genetic testing confirmed autosomal dominant retinitis pigmentosa and provided answers regarding prognosis and family tree considerations. White cane mobility training allowed her to be more independent when traveling. Independent living skills training equipped her to be safer and more autonomous at home. Assistive technology training empowered her to use her digital devices more efficiently to reconnect with friends, family, and the world. A visually impaired clinical counselor helped the patient to work through the process of grieving her vision loss, while involvement in peer support groups allowed her to connect with a new community and recognize future potential. The training and support resources utilized in this case combined to help an individual adapt in response to a challenging diagnosis and prognosis. Such resources should never be overlooked or underestimated in cases of irreversible vision loss.

## 1. Introduction

Retinitis pigmentosa is a retinal neurodegenerative disease which affects approximately 1 in 4000 individuals worldwide, making it the most common inherited retinal dystrophy [1, 2]. This disease can be sporadic or inherited in autosomal dominant, autosomal recessive, x-linked, or mitochondrial fashion [1]. Over 80 genes are known to be associated with nonsyndromic retinitis pigmentosa [1]. Afflicted patients often experience nyctalopia and progressive worsening of peripheral visual fields, in some cases, reaching the level of legal or total blindness [3, 4]. Symptoms are often first recognized in one's teenage years and can progress over decades [5]. Though promising work is being done regarding gene therapy, most cases do not yet have restorative treatments available [6].

Permanent vision impairment has been shown to reduce independence in one's daily activities and self-reported quality of life [7–9]. Further, rates of depression have been found to be notably higher among visually impaired individuals than in the general population [10]. The practice of clinical vision rehabilitation is aimed at providing care and support to those dealing with medically irreversible vision impairment. Comprehensive programs may include the use of tools and strategies to maximize visual function, adaptive training to maintain independence, and psychosocial support to improve quality of life [11].

This report describes the case of a patient found to have an autosomal dominant form of retinitis pigmentosa for which there is no FDA-approved restorative treatment available, though it should be noted that clinical trials are underway to assess gene therapy strategies as potential avenues for vision restoration in such cases. Due to the lack of available medical treatments, this patient underwent a comprehensive vision rehabilitation program to address the many ways in which this diagnosis impacted her independence, quality of life, and mental health.

## 2. Case Presentation

**Figure 1:**
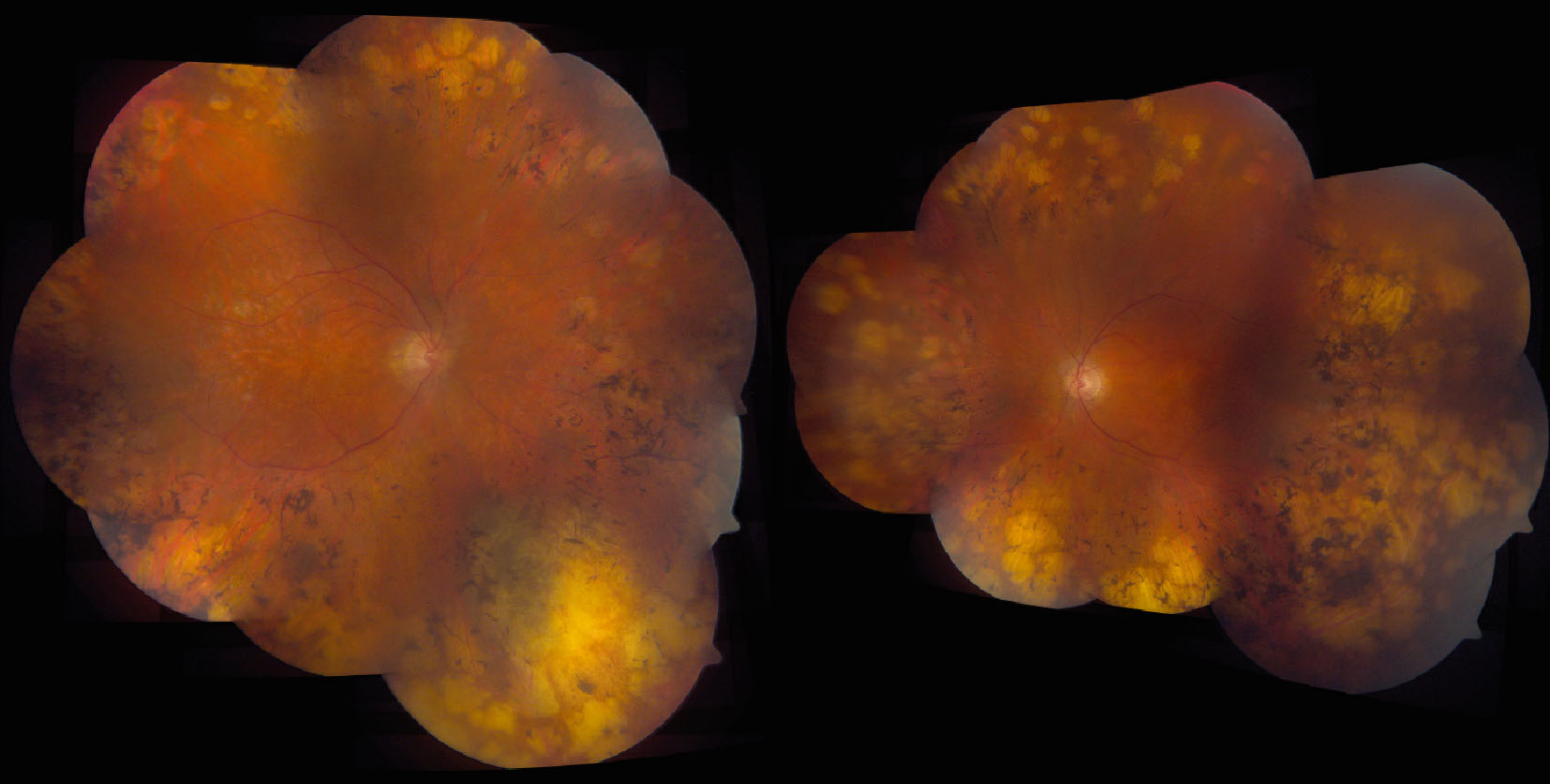
Montage fundus photos of the patient's right and left eyes, each showing characteristic peripheral retinal atrophy and pigmentary changes as well as diffuse vascular attenuation.

**Figure 2:**
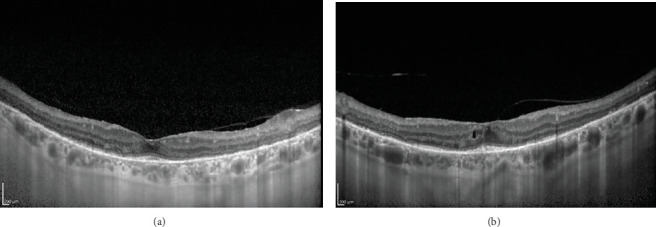
Macular optical coherence tomography scans of the (a) right and (b) left eyes. Each eye demonstrates an epiretinal membrane and ellipsoid zone attenuation with a small amount of outer nuclear layer remaining within the central macula. The left eye shows cystic intraretinal space consistent with the diagnosis of chronic cystoid macular edema.

A 62-year-old female was referred by her ophthalmologist for a low vision rehabilitation evaluation. The patient had been phenotypically diagnosed with retinitis pigmentosa approximately 15 years prior but had never sought genetic confirmation of her diagnosis. The referral also indicated the presence of an epiretinal membrane and chronic cystoid macular edema in both eyes. Montage fundus photos and macular optical coherence tomography scans for the patient are shown in Figures 1 and 2, respectively. The patient's parents were deceased, and she had one daughter and one granddaughter. She denied knowledge of any family ocular history. She had never been evaluated in a low vision rehabilitation clinic before.

The patient's referral to the vision rehabilitation clinic was prompted by her report of worsening peripheral vision, increasing mobility difficulty, and print reading challenges despite relatively stable objective and subjective clinical findings noted by her ophthalmologist. She reported anxiety when traveling outside her home. Depth perception and navigating elevation changes were noted as being particularly troublesome. She also described frustration with having to be so dependent upon her husband, with whom she presented to the initial evaluation, to get around.

The patient was wearing 3-year-old progressive multifocal spectacle lenses during her waking hours and reported using dark gray, polarized filters as needed for relief of light sensitivity outdoors. She did not bring her sunglasses to the visit and reported that they were often not dark enough to relieve glare satisfactorily when walking outside. She had self-discontinued all driving less than 6 months prior to the visit due to a general lack of confidence in her own ability to be a safe driver.

The patient stated that she would like to be more independent in several ways. She noted reluctance to place the burden of transportation on her husband, but her local area did not have accessible public transit or taxi systems. She was not confident in her ability to walk safely in unfamiliar environments, especially when lighting conditions were not bright enough. She was having trouble reading printed materials, specifically recipes and food preparation instructions, through her habitual spectacle lenses and was dependent upon a Kindle (Amazon; Seattle, Washington) or iPhone X (Apple; Cupertino, California) with enlarged text to be able to read digital materials. The patient had reduced the display brightness on her iPhone but still had trouble viewing it comfortably at times.

The patient's best corrected distance visual acuity was found to be 20/40^−2^ in both the right and left eyes, tested individually. A reverse contrast display was subjectively noted to be more comfortable to look at, but without measurable improvement in acuity. Binocular, continuous near-text reading acuity using the MNREAD Acuity Chart (Precision Vision; Woodstock, Illinois) was 20/50 (Snellen equivalent) under full room illumination and 20/40 (Snellen equivalent) with an additional daylight fluorescent task light directed onto the reading card from 40 cm away.

Monocular confrontation visual fields were noted as severely constricted in all quadrants, in each eye. Binocular contrast sensitivity was measured at 1.44 log units, which equates to moderate impairment, using the MARS Perceptrix contrast chart (The MARS Perceptrix Corporation; Chappaqua, New York).

A filter demonstration was completed in full room illumination using an Eschenbach Tint Kit (Eschenbach Optik of America Inc; Danbury, Connecticut). This started with the determination of the approximate transmission percentage preferred by the patient for best visual comfort (8%–15%) and concluded with the demonstration of all filter color options within this range. Ultimately, the 11% transmission polarized gray filters provided the best subjective comfort for this patient in full room illumination.

**Figure 3:**
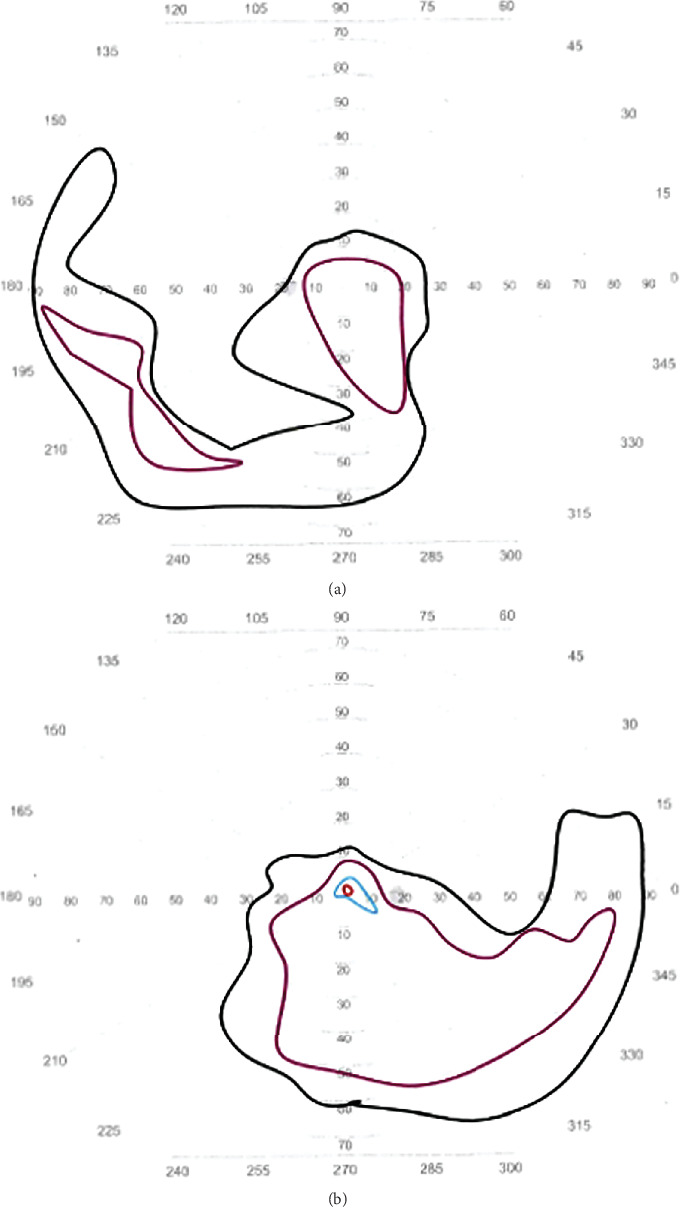
Goldmann visual field results for the (a) left and (b) right eyes. Isopters depicted include V4e (black), III4e (purple), I4e (blue), and I2e (red). The results depict irregular loss of peripheral vision to all targets with some sparing to the V4e and III4e in the inferior hemisphere of each eye.

Kinetic full-field perimetry using V4e, III4e, I4e, and I2e targets was completed with the Haag-Streit Octopus 900 kinetic perimeter (Haag-Streit USA; Mason, Ohio). The patient fixated well with each eye, and the test results showed irregular loss of peripheral vision, more notable in the left eye than in the right (Figure 3).

An updated progressive multifocal spectacle prescription was provided, along with a product catalog (LS&S, LLC; Buffalo, New York) for ordering 11% gray polarized fit-over filters for use as needed. An adjustable desktop arm-based task lamp was also recommended from the catalog for print reading at home. The patient was encouraged to shine the lamp directly onto the page from close proximity to maximize page illumination and at an angle directed away from her eyes to minimize undue glare.

The patient was given a brief demonstration of some accessibility features on her iPhone, including Smart Invert, Magnifier, and the Accessibility Shortcut. The plan was to expand upon this introduction through referral for more comprehensive assistive technology training.

An order for an at-home genetic testing kit was placed through the My Retina Tracker inherited retinal disease panel program, made free through the support of the Foundation Fighting Blindness (Columbia, Maryland). Results were to be conveyed to the patient through the program's genetic counselors.

Several additional referrals were made following the evaluation visit. The patient was referred to the state of Tennessee's blind and independent living skills training program for an in-home visit. The order asked specifically for iPhone accessibility training, strategies for improved independence in completing activities of daily living, and safe mobility skills. To more comprehensively address her mobility needs, the patient was also referred to a local Certified Orientation and Mobility Specialist for white cane mobility training.

To assist with the patient's psychological adaptation to her impairment, she was referred to a local counselor who is completely blind and helps to support many individuals who are dealing with irreversible vision loss. Finally, the patient was invited to join a bimonthly support group series hosted by the clinic to help connect her with a community of individuals with visual difficulties. She has since been a regular attendee of these meetings.

Approximately 2 months after her evaluation visit, the patient attended a support group meeting with her husband. The patient was carrying a folded white mobility cane and utilizing proper sighted guide technique with her right hand grasping her husband's left arm just above the elbow. She stated that she had met with the Certified Orientation and Mobility Specialist one time to assess her needs, take measurements for a cane, and learn sighted guide alongside her husband. The patient had also been in touch with the counselor and had a scheduled meeting with him. Finally, the patient had sent in her saliva sample to the My Retina Tracker inherited retinal disease testing program.

At the next bimonthly meeting, the patient reported that three white cane training sessions and two counseling visits had taken place. She was beginning to travel independently in her local area with the cane and reported growth in self-confidence. She noted that her counseling visits had been helping a great deal and that she planned to attend the next local National Federation of the Blind meeting to meet more people who had been diagnosed with retinitis pigmentosa. The patient had met with the state-based independent living skills program to discuss her needs and had scheduled an in-home training visit with them.

At the next support group meeting, the patient reported that she was continuing her white cane training and counseling visits. She stated that her newfound mobility confidence and independence had been “liberating” for her and that she was feeling less like a burden to her husband.

The patient also reported that the independent living skills program had worked twice with her inside her home. They had helped to alter her kitchen lighting by increasing luminance and reorienting some lighting directions. These steps had improved her ability to use kitchen appliances and cook more independently. She reported that some helpful talking devices, such as a kitchen timer and watch, had been provided to her as well. She could set and run the kitchen timer with a set of buttons which provided auditory output with each push, and her new watch could tell her the current time and date at the push of a button. Her trainer had also helped to connect the patient with a local paratransit agency, which would allow her to arrange transportation within her community without needing to rely on her husband.

At the insistence of her independent living skills trainer, the patient had met with a Technology Access Program specialist and downloaded some helpful free applications for her iPhone. These included Seeing AI, which uses the device's camera to convert printed material into auditory output, and Be My Eyes, which pairs a visually impaired user with a sighted volunteer in a video chat to provide descriptions of the user's environment. The patient was using both applications in the kitchen, with Seeing AI helping her to access printed recipes and Be My Eyes allowing her to check expiration dates on food products and set her oven and stove dials accurately. She had also implemented the Smart Invert contrast inversion feature on her iPhone and noted improved efficiency in reading text messages and phone settings menus since her training.

The patient's genetic testing results had shown a causative heterozygous variant in the *PRPH2* gene (c.828+3A>T, intronic). She noted that a telephone discussion with the genetic counselor had been helpful in educating her on this finding, its implications for her family, and the potential for future therapies.

## 3. Discussion

This patient presented with a diagnosis which had been made 15 years prior, but a recent worsening in symptom severity had compromised her independence. A comprehensive rehabilitation plan, aimed at addressing her list of vision-related needs, altered her outlook dramatically.

The uncovering of a causative variant in *PRPH2* gave insight into the inheritance pattern of her retinal condition and provided hope for effective therapeutic advances in the future. *PRPH2* codes for Peripherin 2, a transmembrane glycoprotein found in photoreceptor outer segments [12]. *PRPH2* variants have been implicated in numerous inherited retinal diseases, including autosomal dominant retinitis pigmentosa [12]. The genetic counselor was able to inform the patient and her only child of the familial implications of an autosomal dominant inheritance pattern. The patient's daughter has since been offered free familial genetic testing through the My Retina Tracker program.

White cane mobility training was the key in this patient's journey to greater independence. However, referral can sometimes be difficult to accept despite the immense potential this training holds. Some patients associate the white cane with a social stigma or increased vulnerability, while others may view the use of a white cane as a step away from independence rather than toward it [13]. Users may be viewed as dependent upon the white cane, but proper use of this tool can actually convey a sense of independence [14]. This patient described her newfound confidence and the independence gained through using her white cane, as she was no longer dependent upon her husband to get around safely.

In-home independent living skills training and assistive technology strategies were also pieces in this patient's rehabilitative puzzle. She was now able to be more independent at home, thanks to improved lighting, adaptive tools, and strategies learned. Further, she learned to utilize the built-in accessibility features of her iPhone and downloaded some helpful applications.

A combination of one-on-one counseling and introduction to a community of individuals going through similar struggles allowed this patient to realize that she was not alone and that there is hope to be found in a life with vision loss.

Table 1 summarizes the patient's needs upon presentation to the vision rehabilitation clinic, the interventions involved in addressing these needs, and the postintervention functional improvements resulting from these interventions.

## 4. Conclusion

Prescription of a refractive correction and filters may be important when caring for patients with irreversible vision impairment, but these steps alone do not represent comprehensive patient care. An individualized and comprehensive rehabilitation plan should be implemented to address a patient's needs, from independent living skills to psychosocial adjustment. This case demonstrates the profound impact such an approach can have. No identifiable health information was included in this case report.

## Figures and Tables

**Table 1:** Summary of comprehensive rehabilitation plan components and their effects on the patient's stated goals and needs at the time of referral.

**Preintervention**	**Interventions**	**Postintervention**
Print reading difficulty	Progressive multifocal Rx Home lighting updates Task lighting implementation	Improved print reading ability

Sunlight glare sensitivity	11% transmission gray fit-over filters	Improved visual comfort in bright light conditions

Difficulty using digital devices	Technology Access Program	Utilization of device accessibility features and accessible applications Improved ability to read recipes and labels using digital devices

Mobility dependence on husband Trouble with navigation, crowds, and elevation changes	White cane mobility training	Improved confidence and mobility independence Proper sighted guide skills

Transportation dependence	Paratransit services	Transportation independence

Feelings of isolation	One-on-one counseling Support group	Community connection Improved mental health status Connection with National Federation for the Blind

Uncertainty regarding inheritance and prognosis	Genetic testing (*PRPH2*)	Prognostic information Autosomal dominant inheritance awareness Potential clinical trial eligibility

## Data Availability

Data sharing is not applicable to this article as no new data were created or analyzed in this study.
